# Provincial Schools

**Published:** 1907-09-07

**Authors:** 


### PROVINCIAL SCHOOLS.

*The University of Birmingham (Faculty of Medicine).*—The composition fee for the whole course of lectures and instruction at the University is £85, besides which there is a composition fee for attendance on the medical and surgical practice and the clinical lectures at both hospitals of £42, making a total of £127. Dean: Professor Gilbert Barling, F.R.C.S. The Winter Session 1907-8 commences on Tuesday, October 1.

*University College, Cardiff.*—Since it was founded, in 1883, University College, Cardiff, has prepared students for the Preliminary Scientific examination of the University of London, and for the corresponding examinations of other licensing bodies. In 1893, Chairs of Anatomy and Physiology and a Lectureship in Materia Medica and Pharmacy were established, making it possible to spend three out of the five years of the medical curriculum at this school.

Arrangements have been made with the managing committee of the Cardiff Infirmary to give students of the college the privilege of attending this hospital, which contains 180 beds.

The composition fee for the three years' course of study for the Preliminary Scientific and Intermediate Examination for the London M.B. is £57 10s.

*University of Durham (College of Medicine, Newcastle-upon-Tyne).*—A new wing has been added to the College of Medicine to accommodate the departments of physiology and bacteriology. It also contains a students' gymnasium and a set of students' union rooms. The New Royal Victoria Infirmary, containing over 400 beds, was recently opened by H.M. the King. In the new infirmary adequate accommodation will be provided for the study of the various special subjects in addition to the ordinary clinical work.

The composition fee for the complete course of lectures is 72 guineas, and for the medical and surgical practice of the hospital 25 guineas.

*The University of Leeds (School of Medicine).*—Clinical instruction is given in the General Infirmary, which contains 480 beds, and is amply provided with special departments.

Students of the University also have access for instruction to the Leeds Public Dispensary, the City Fever Hospitals, the Hospital for Women and Children, the Leeds Maternity Home, and the West Riding Asylum at Wakefield.

The School of Medicine, which is particularly well equipped in every way, is separated from the Infirmary by the width of a street only, an arrangement which facilitates attendance at both institutions.

*The University of Liverpool* (*Faculty of Medicine*).—Students are prepared for the degrees of the University of Liverpool, or may study for the degrees and qualifications of various other licensing bodies.

The laboratories are modern and very well equipped, and the facilities for instruction are most complete.

Courses of instruction in tropical diseases can be obtained at the Liverpool School of Tropical Medicine. Fellowships, scholarships, and prizes of over £800 are awarded annually.

The Clinical School of the University now consists of four general hospitals—the Royal Infirmary, the David Lewis Northern Hospital, the Royal Southern Hospital, and the Stanley Hospital; and of five special hospitals—the Eye and Ear Infirmary, the Hospital for Women, the Infirmary for Children, St. Paul's Eye and Ear Hospital, and St. George's Hospital for Skin Diseases. These hospitals contain in all a total of 1,127 beds. The organisation of these hospitals to form one teaching institution provides the medical student and the medical practitioner with a field for clinical education and study which is unrivalled in extent in the United Kingdom.

*The Victoria University of Manchester* (*Faculty of Medicine*).—The medical department of the University is particularly well provided with facilities for teaching the preliminary subjects—namely, anatomy, physiology, pathology, and materia medica.

Clinical instruction is provided at the Manchester Royal Infirmary, which contains 290 beds, and also has large out-patient and casualty departments. Associated with the Infirmary is a Convalescent Hospital at Cheadle, containing 136 beds. Women students are admitted to the practice of the Infirmary on the same terms as men.

*Oxford University.*—One of the chief events in the recent history of Oxford University has been the revival of the Medical School. Admirable arrangements now exist for instruction in the preliminary medical sciences; while a certain proportion of the clinical work can be done, if desired, in the wards of the Radcliffe Infirmary, previous to taking out the full course in the metropolis. Clinical lectures are given in medicine and surgery, and demonstrations in pathology, surgical anatomy, and medical and surgical diagnosis are given also. In order to obtain the Oxford medical degree it is necessary to be a graduate in Arts of the University.

*The University of Sheffield.*—The Winter Session 1907-1908 begins on Wednesday, October 2. The University is within easy reach of the various hospitals with which it is connected for clinical purposes. These are as follows: The Royal Infirmary, contains 255 beds, with an annual average number of over 3,800 in-patients, over 8,000 out-patients, and over 21,000 casualties. The Royal Hospital, with 165 beds, and an annual number of 2,500 in-patients, over 7,000 out-patients, and over 14,000 casualties. The Jessop Hospital for Diseases of Women, with eighty beds, nearly 500 in-patients, and over 2,000 out-patients; also a Maternity Department, with over 250 in-patients per annum, and over 700 out-patient cases attended. Special courses on fevers are held at the City Fever Hospitals (547 beds), and on Mental Diseases at the South Yorkshire Asylum (1,610 beds).

*Fees.*—The Composition Fee of £80 is payable in three instalments, namely, £24 at commencement of first year of study; £28 at commencement of second year of study; £28 at commencement of third year of study. The Composition Fee does not include medical and surgical hospital practice, clinical lectures, practical instruction in mental diseases, diseases of women, and infectious diseases. Composition fee for Medical and Surgical Hospital Practice for the full period required by the Examining Boards: If paid in one sum at commencement of Hospital Practice, £36 15s.; or, if paid in two sums of £18 18s., one on beginning Hospital Practice, the other twelve months later, £37 16s.

